# Improving Lung Cancer Screening Selection: The HUNT Lung Cancer Risk Model for Ever-Smokers Versus the NELSON and 2021 United States Preventive Services Task Force Criteria in the Cohort of Norway: A Population-Based Prospective Study

**DOI:** 10.1016/j.jtocrr.2024.100660

**Published:** 2024-03-05

**Authors:** Olav Toai Duc Nguyen, Ioannis Fotopoulos, Maria Markaki, Ioannis Tsamardinos, Vincenzo Lagani, Oluf Dimitri Røe

**Affiliations:** a Department of Clinical Research and Molecular Medicine, Norwegian University of Science and Technology, Trondheim, Norway; b Levanger Hospital, Nord-Trøndelag Hospital Trust, Cancer Clinic, Levanger, Norway; c Department of Computer Science, University of Crete, Voutes Campus, Heraklion, Greece; d Institute of Applied and Computational Mathematics, Heraklion, Greece; e JADBio Gnosis Data Analysis (DA) S.A., Science and Technology Park of Crete (STEP-C), Heraklion, Greece; f Biological and Environmental Sciences and Engineering Division (BESE), King Abdullah University of Science and Technology (KAUST), Thuwal, Saudi Arabia; g Saudi Data and Artificial Intelligence Authority (SDAIA)–KAUST Center of Excellence in Data Science and Artificial Intelligence, Thuwal, Saudi Arabia; h Institute of Chemical Biology, Ilia State University, Tbilisi, Georgia; i Clinical Cancer Research Center and Department of Clinical Medicine, Aalborg University Hospital, Aalborg, Denmark

**Keywords:** Lung cancer screening, Screenee selection, Risk prediction models, CONOR, HUNT

## Abstract

**Background:**

Improving the method for selecting participants for lung cancer (LC) screening is an urgent need. Here, we compared the performance of the Helseundersøkelsen i Nord-Trøndelag (HUNT) Lung Cancer Model (HUNT LCM) versus the Dutch-Belgian lung cancer screening trial (Nederlands-Leuvens Longkanker Screenings Onderzoek (NELSON)) and 2021 United States Preventive Services Task Force (USPSTF) criteria regarding LC risk prediction and efficiency.

**Methods:**

We used linked data from 10 Norwegian prospective population-based cohorts, Cohort of Norway. The study included 44,831 ever-smokers, of which 686 (1.5%) patients developed LC; the median follow-up time was 11.6 years (0.01–20.8 years).

**Results:**

Within 6 years, 222 (0.5%) individuals developed LC. The NELSON and 2021 USPSTF criteria predicted 37.4% and 59.5% of the LC cases, respectively. By considering the same number of individuals as the NELSON and 2021 USPSTF criteria selected, the HUNT LCM increased the LC prediction rate by 41.0% and 12.1%, respectively. The HUNT LCM significantly increased sensitivity (*p* < 0.001 and *p* = 0.028), and reduced the number needed to predict one LC case (29 versus 40, *p* < 0.001 and 36 versus 40, *p* = 0.02), respectively. Applying the HUNT LCM 6-year 0.98% risk score as a cutoff (14.0% of ever-smokers) predicted 70.7% of all LC, increasing LC prediction rate with 89.2% and 18.9% versus the NELSON and 2021 USPSTF, respectively (both *p* < 0.001).

**Conclusions:**

The HUNT LCM was significantly more efficient than the NELSON and 2021 USPSTF criteria, improving the prediction of LC diagnosis, and may be used as a validated clinical tool for screening selection.

## Introduction

Lung cancer (LC) is the leading cause of cancer mortality worldwide.^1^ Despite decades of research, the prognosis of LC is still poor. The general 5-year overall survival is as low as 20% and is owing to the lack of early detection and insufficient effect of late-stage treatments.^2^ Early detection of LC is, therefore, crucial to improve the prognosis.^2^

The U.S. National Lung Screening Trial (NLST) and European Nederlands-Leuvens Longkanker Screenings Onderzoek (NELSON) studies reported that computed tomography (CT) screening could reduce LC mortality by 20% and 24%, respectively.^3^^,^^4^ Both studies used age and smoking criteria to identify high-risk individuals to be screened. However, only a quarter of all who develop LC fulfill the NLST criteria,^5^ meaning that most individuals developing LC would be ineligible for CT screening on the basis of these criteria. To improve the sensitivity, the U.S. Preventive Strategy Task Force (USPSTF) recommended the inclusion criteria in 2013 to age 55 to 80 years old, greater than or equal to 30 pack-years, and currently smoking or quit smoking earlier than 15 years.^6^ In 2021, the USPSTF recommended further expansion of the age range and reduction of the pack-year eligibility criteria (age 50–80 years old, ≥20 pack-years, and currently smoking or quit smoking <15 years).^7^ Recently, a study reported that the 2021 USPSTF criteria seem to be more cost-effective compared with the 2013 USPSTF criteria.^8^

There is growing evidence that risk-based approaches are more sensitive and effective in identifying individuals with a high risk of LC compared with categorical clinical cutoff approaches such as the NLST, NELSON, and USPSTF criteria.^9^, ^10^, ^11^, ^12^, ^13^ In previous work, we developed a risk model and online calculator, the HUNT Lung Cancer Model (HUNT LCM), on the basis of eight variables (sex, age, body mass index, pack-years, number of cigarettes per day, quit time in years, hours of daily indoors smoke exposure and history of daily cough in periods through the year).^11^ The HUNT LCM was developed from the population-based prospective study HUNT2 of 65,018 individuals and was externally validated on the large prospective Norwegian population Cohort of Norway (CONOR) of ever-smokers, revealing a predictive power measured by the concordance (C-) index of 0.879 and an area under the receiver operating characteristic curve for LC diagnosis within 6 years of 0.87.^11^ A 6-year LC risk was used in the original work developing the HUNT LCM to allow comparisons with other validated models, for instance, the Prostate, Lung, Colorectal, and Ovarian Cancer Screening model 2012 (PLCO_m2012_) that used 6-years risk.^13^ The model was revealed to be considerable more predictive and effective compared with the NLST criteria.^11^ To be able to inform health policymakers, it is important to compare the performance of validated risk prediction models with current and generally accepted criteria.

In the present work, we aim to evaluate the HUNT LCM against the NELSON and 2021 USPSTF criteria regarding LC prediction and efficiency in a Norwegian, prospective cohort study (CONOR).

## Material and Methods

### The Prospective Cohort (CONOR)

The individuals included were all from the pan-Norwegian prospective population-based study, CONOR. CONOR is already described in previous work^11^; briefly, it consists of 11 regional prospective population-based studies, comprising 180,534 individuals older than 19 years old in which all clinical variables were collected at inclusion in the studies. All the CONOR substudies had the same variables as used for the HUNT LCM.^11^^,^^14^ The HUNT2 participants in CONOR were excluded (n = 65,018) because the HUNT2 cohort was used for the development of the HUNT LCM. The remaining 115,516 were screened for inclusion in the study. The HUNT LCM applies only to ever-smokers. After excluding never-smokers (n = 34,746), those with a previous history of LC (n = 86), and those with one or more missing variables (n = 35,853), an ever-smokers cohort with complete data were included in the final analysis (n = 44,831) (Supplementary Fig. 1). This cohort was linked with the Norwegian Cancer Registry, in which the LC diagnosis date was identified, and with the Death Registry, in which the death date was registered. Individuals who developed LC within the whole follow-up period and within 6 years were identified. International Classification of Diseases 7 162.1 and International Classification of Diseases 10 C34.0-C34.9 were used to identify participants that developed LC. Those who developed other types of cancer were not excluded. Individuals who died were censored at the time of death.

### Ethics

Participants included in the CONOR all gave their written consent according to the Helsinki Declaration. The Norwegian Data Inspectorate and the Regional Committees for Medical Research Ethics approved each individual study.

### Univariate Analysis

The association between LC and each of the eight clinical variables was assessed by univariate analysis through Pearson linear correlation (numerical variables) or the Wilcoxon test (categorical variables). The distribution of the covariates in the whole cohort (n = 80,770) before and after filtering in the samples without missing values (n = 44,831) were compared using the Kolmogorov-Smirnov test (continuous variables) and chi-square test (categorical variables).

### Model Comparisons

All model comparisons aimed to evaluate performance in predicting the risk of being diagnosed with LC 6 years after inclusion in the CONOR study. The performance of the HUNT LCM in predicting LC diagnosis was compared against the NELSON and 2021 USPSTF screening eligibility criteria. Specifically, the HUNT LCM was used to rank patients according to their 6-year risk of diagnosis. The patients with a risk higher than a given threshold were predicted as possible LC future diagnosed cases. The NELSON and 2021 USPSTF screening eligibility criteria are fixed, whereas a model risk eligibility criteria such as the HUNT LCM can be adjusted, for instance, according to the capacity of the health system. For a fair comparison, we used a risk threshold selecting the same number to screen as the NELSON criteria and 2021 USPSTF criteria as a benchmark. Given this threshold, several metrics of predictive performance are presented including sensitivity, specificity, positive predictive value (PPV), negative predictive value, and number needed to “screen” (NNS)—in this case, the numbers needed to predict one clinically diagnosed LC in 6 years. We used NNS as a metric to evaluate an eligibility criteria’s efficiency. The statistical significance for differences in these metrics was assessed through a nonparametric, permutation-based test.

To investigate the predictive performance of the HUNT LCM when excluding the older population, we performed the analysis for the same number of participants as selected by the criteria but used the upper age criterion of the NELSON and 2021 USPSTF criteria as cutoff, younger than 75years and younger than 81 years of age, respectively. We also investigated the predictive performance of the HUNT LCM with an age cutoff of 50 to 80 years.

Moreover, we investigated and compared overall survival between the subcohorts selected by the HUNT LCM, NELSON, and 2021 USPSTF criteria. The survival was calculated as the median overall survival from LC diagnosis in the cases. Kaplan-Meier curves were used to visualize survival and log-rank tests to evaluate the statistical significance. For all analyses, *p* less than 0.05 was used as the statistical significance level. The R version 4.2.1 (2022-06-23, R Core Team, Vienna, Austria) was used to perform the analyses.

Finally, the predictive performance of the HUNT LCM was also tested using the top 16th percentile computed on the ever-smokers in the HUNT2 cohort (risk scores >0.98% risk in 6 years) according to recommendations from Royston et al.^11^^,^^15^ (see Supplementary Methods). This threshold was applied in CONOR to stratify patients into high- and low-risk categories according to the HUNT LCM.

## Results

### The CONOR Ever-Smokers Data Set

The CONOR cohort included 44,831 ever-smokers, of which 686 (1.5%) patients developed LC during the whole follow-up (Supplementary Fig. 1). The median follow-up time was 11.6 years (0.01–20.8 years) with a total of 561,405 person-years.

The number of LCs occurring within 6 years from inclusion was 222 (0.5%) (Supplementary Fig. 1) and the age distribution at the time of inclusion in the CONOR study of all the participants that developed LC is presented in Supplementary Table 1. The clinical variables except body mass index, were significantly associated with LC occurrence in univariate analysis (Table 1). The results below were calculated within the 6-year follow-up frame after inclusion in the CONOR study.

**Table 1:** Descriptive Statistics for the External Study Population CONOR at Inclusion

Clinical Variables	CONOR Ever-Smokers			
	N	No Lung Cancer n = 44,609	Lung Cancer n = 222	*p* Value
Sex	44,831			<0.001
Female		23,058 (51.7)	77 (34.7)	
Male		21,551 (48.3)	145 (65.3)	
Age	44,831			<0.001
Mean (SD), range		48.80 (13.11), 24.7–94.5	64.96 (10.13), 32.0–86.5	
Pack-year	44,831			<0.001
Mean (SD), range		13.72 (12.20), 0.05–168.0	28.41 (16.72), 1.0–120.0	
Daily cough parts of the y	44,831			<0.001
No		34788 (78.0)	130 (58.6)	
Yes		9821 (22.0)	92 (41.4)	
Indoor smoke exposure in h	44,831			<0.001
Mean (SD), range		2.55 (4.09), 0.0–24.0	4.14 (4.83), 0.0–20.0	
Quit time in y	44,831			<0.001
Mean (SD), range		6.48 (10.19), 0.0–69.0	4.19 (9.06), 0.0–45.0	
Cigarettes daily	44,831			<0.001
Mean (SD), range		11.97 (7.10), 1.0–99.0	14.14 (7.77), 1.0–60.0	
Body mass index	44,831			0.309
Mean (SD), range		25.70 (4.01), 11.87–53.54	25.42 (4.43), 13.74–40.98	

*Note:* All patients were ever-smokers. The *p* value of the statistical association of each variable with a lung cancer diagnosis within 6 years. All values are n (%) unless otherwise specified.

CONOR, Cohort of Norway.

### Predictive Performance Assuming Equal Number of Participants—HUNT LCM Versus NELSON and 2021 USPSTF Criteria

**Figure 1:**
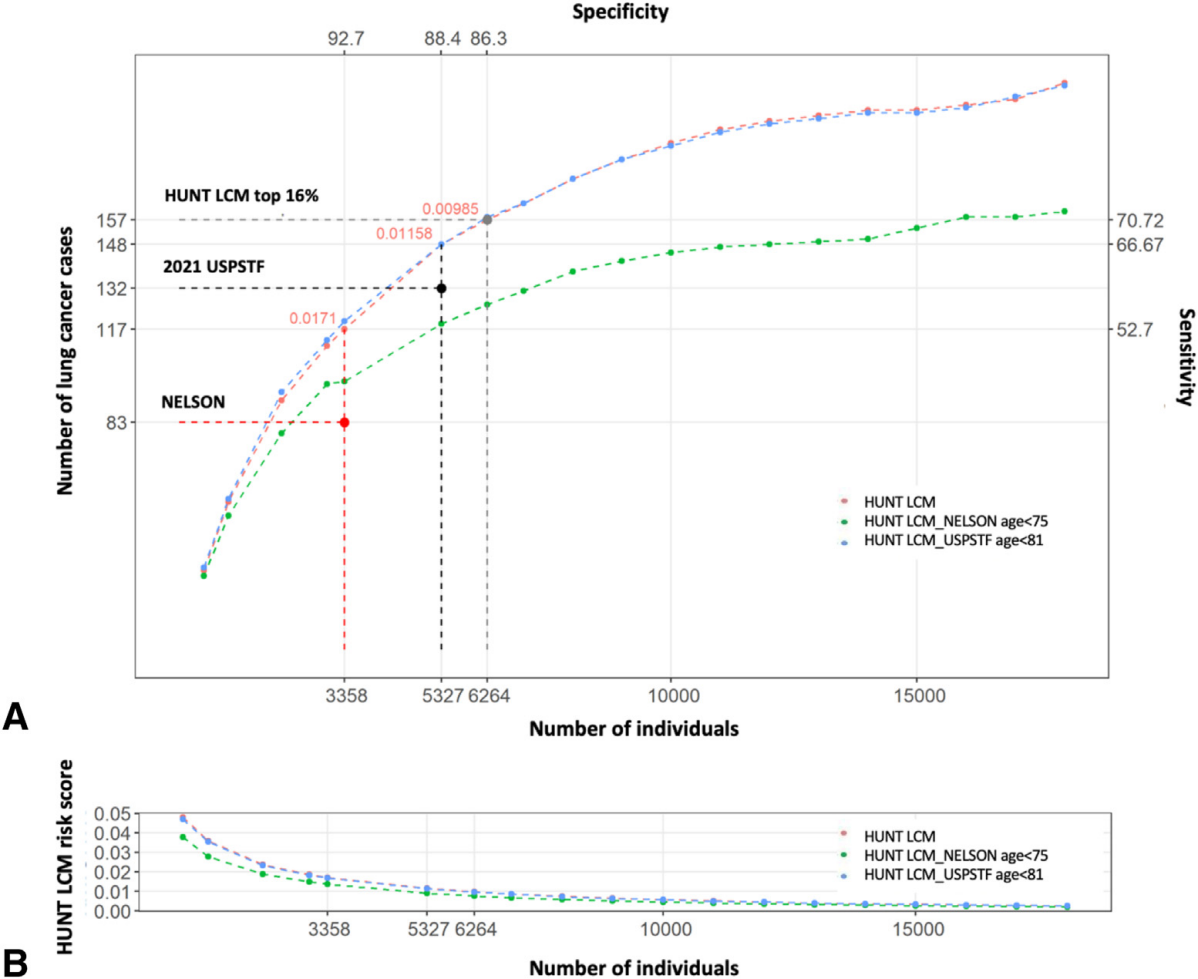
Predictive performance of the HUNT LCM compared against the NELSON and 2021 USPSTF criteria on the CONOR ever-smoker cohort. (*A*) Comparison of the predictive performance of the HUNT LCM in 6 years by decreasing risk scores (x-axis) against the NELSON and 2021 USPSTF criteria on the CONOR ever-smoker cohort (n = 44831) (red line). The NELSON number of participants by HUNT LCM had a risk score greater than 1.71% in 6 years. The 2021 USPSTF number of participants by HUNT LCM had a risk score greater than 1.16% in 6 years. The HUNT LCM top 16th risk score in the HUNT2 represents the absolute cutoff 0.985% risk in 6 years. Predictive performance analysis of the HUNT LCM using the upper age criterion of the NELSON and 2021 USPSTF criteria including only individuals age younger than 75 and younger than 81 years, respectively. (B) Decreasing HUNT LCM risk score in CONOR ever-smokers from left to right. HUNT LCM risk score 0.01 represents 1% risk of lung cancer in 6 years, 0.02 represents 2% risk, and others. HUNT LCM, HUNT Lung Cancer Model; CONOR, Cohort of Norway; NELSON, Nederlands-Leuvens Longkanker Screenings Onderzoek; USPSTF, United States Preventive Services Task Force.

The NELSON and 2021 USPSTF criteria identified 3275 and 5195 patients as high-risk for LC, respectively. Within these two subcohorts, 83 (2.5%) and 132 (2.5%) patients for the NELSON and 2021 USPSTF criteria, respectively, were diagnosed with LC. The corresponding sensitivities were 37.4% and 59.5%, respectively (Fig. 1, Table 2 and Supplementary Table 2). Consequently, under the NELSON and 2021 USPSTF criteria, 139 (62.6%) and 90 (40.5%) of future LC cases in 6 years were excluded. In contrast, the HUNT LCM identified 117 (3.5%) and 148 (2.5%) true positives with respective sensitivities of 52.7% (*p* < 0.001) and 66.7% (*p* = 0.025), and increased LC detection rate of 41.0% and 12.1% compared with the NELSON and 2021 USPSTF criteria, respectively (Fig. 1, Table 2 and Supplementary Table 2). The specificities were similar between the HUNT LCM and the two criteria—that is, 92.7% versus 92.7% (NELSON, *p* = 0.63) and 88.4% versus 88.4% (2021 USPSTF, *p* = 0.79).

**Table 2:** Comparison of the HUNT LCM Against the NELSON Criteria

NELSON/HUNT LCM	CONOR Ever-Smokers			
	Lung Cancer (n = 222)	No Lung Cancer (n = 44,609)	Total (N = 44,831)	Predictive Value
**NELSON**				
Criteria positive	83 TP (2.5%)	3275 FP (97.5%)	3358	PPV 2.5%
Criteria negative	139 FN (0.3%)	41,334 TN (99.7%)	41,473	NPV 99.7%
Sensitivity	37.4%			
Specificity		92.7%		
**HUNT LCM**				
Criteria positive	117 TP (3.5%)	3241 FP (96.5%)	3358	PPV 3.5%^a^
Criteria negative	105 FN (0.3%)	41,368 TN (99.8%)	41,473	NPV 99.8%^a^
Sensitivity	52.7%^a^			
Specificity		92.7%^b^		

CONOR, Cohort of Norway; HUNT LCM, HUNT Lung Cancer Model; NELSON, Nederlands-Leuvens Longkanker Screenings Onderzoek; FN, false-negative; FP, false-positive; NPV, negative predictive value; PPV, positive predictive value; TN, true negative; TP, true positive.

*Note:* The comparison is performed by considering the number of individuals selected by NELSON criteria—that is, n equals 3358 on the CONOR ever-smokers cohort (n = 44831). The NELSON criteria include the following: age between 50 to 74 years old, greater than 15 cigarettes per day for more than 25 years, or greater than10 cigarettes per day for more than 30 years, quit smoking less than or equal to10 years.

a *p* Value less than 0.01, versus NELSON.

b *p* Value greater than 0.05, versus NELSON.

The comparisons above impose no upper age limit in the HUNT LCM. However, both the NELSON and 2021 USPSTF criteria impose upper age limits, including individuals younger than ages 75 and 80 years, respectively. The predictive performance of HUNT LCM was compared with the two sets of criteria when the same number of screenees were selected, and upper age limits were imposed. Under these conditions, the HUNT LCM identified more true positives (98 versus 83) and with a significantly higher sensitivity (57.0% versus 48.3%, *p* = 0.019) compared with the NELSON criteria (Supplementary Table 3). Similarly, for the 2021 USPSTF criteria, the HUNT LCM identified more true positives (148 versus 132) and had a significantly higher sensitivity (67.0% versus 59.7%, *p* = 0.009) (Supplementary Table 4). The HUNT LCM was more eligible to individuals in the age interval of 70 to 79 years and less in the age interval of 50 to 59 years compared with the NELSON (Supplementary Table 5) and 2021 USPSTF criteria (Supplementary Table 6) when equal numbers of individuals were considered. Furthermore, the same comparison was performed but with an age cutoff of 50 to 80 years, exhibiting similar results in which the HUNT LCM identified more true positives (121 versus 83) and with a significantly higher sensitivity (60.2% versus 41.3%, *p* < 0.001) compared with the NELSON criteria (Supplementary Table 7). Similarly, for the 2021 USPSTF criteria, the HUNT LCM identified more true positives (148 versus 132) and had a significantly higher sensitivity (73.6% versus 65.7%, *p* = 0.01) (Supplementary Table 8).

### NNS to Predict One Clinically Diagnosed LC

**Figure 2:**
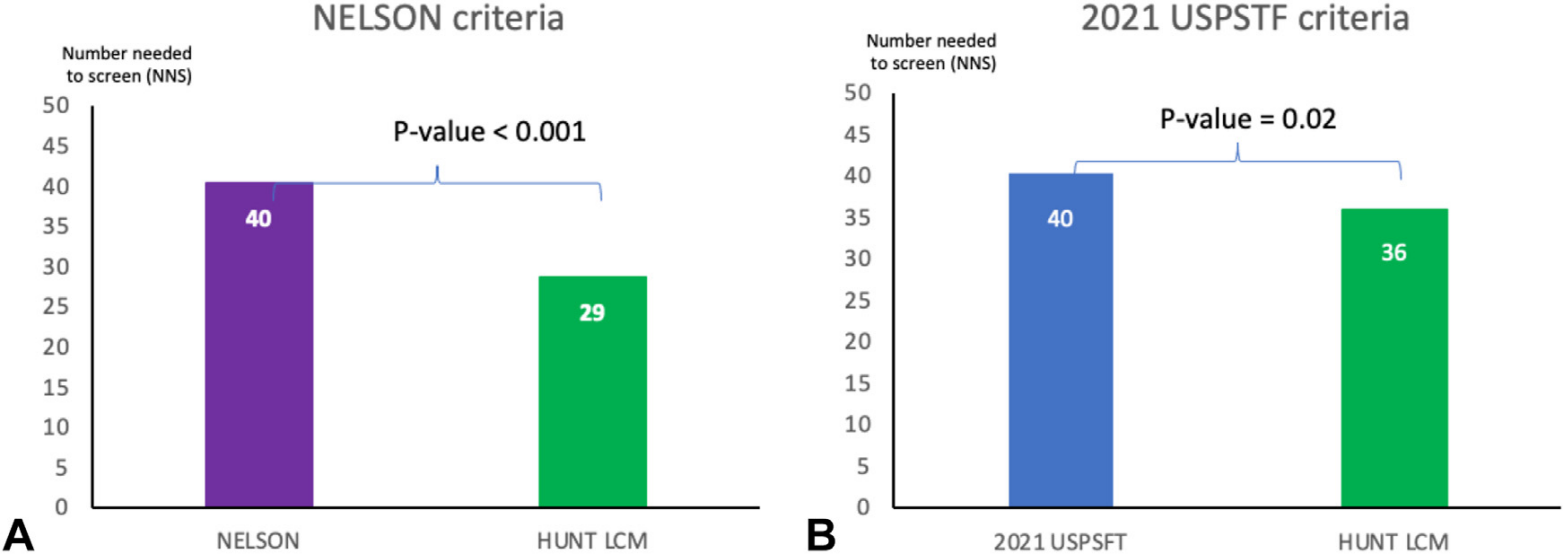
NNS to identify one case of lung cancer in CONOR. NNS computed when screening the same number of people selected by (*A*) NELSON criteria (NELSON: age between 50 to 74 years old, >15 cigarettes per day for >25 years or >10 cigarettes per day for >30 years, quit smoking ≤10 years) and (*B*) 2021 USPSTF criteria (2021 USPSTF: age between 50 to 80 years old, at least 20 pack-years, and currently smoking or quit smoking <15 years). HUNT LCM, HUNT Lung Cancer Model; NSS, number needed to screen; CONOR, Cohort of Norway; NELSON, Nederlands-Leuvens Longkanker Screenings Onderzoek; USPSTF, United States Preventive Services Task Force.

The NNS of the HUNT LCM resulting from the analysis of the same number of people as selected by the NELSON and 2021 USPSTF criteria, respectively, revealed statistically significant lower NNS to identify one LC case compared with the NELSON (29 versus 40, *p* < 0.001) and 2021 USPSTF criteria (36 versus 40, *p* = 0.02) (Fig. 2).

### Survival Analysis Assuming Equal Number of Participants—HUNT LCM Versus NELSON and 2021 USPSTF Criteria

**Figure 3:**
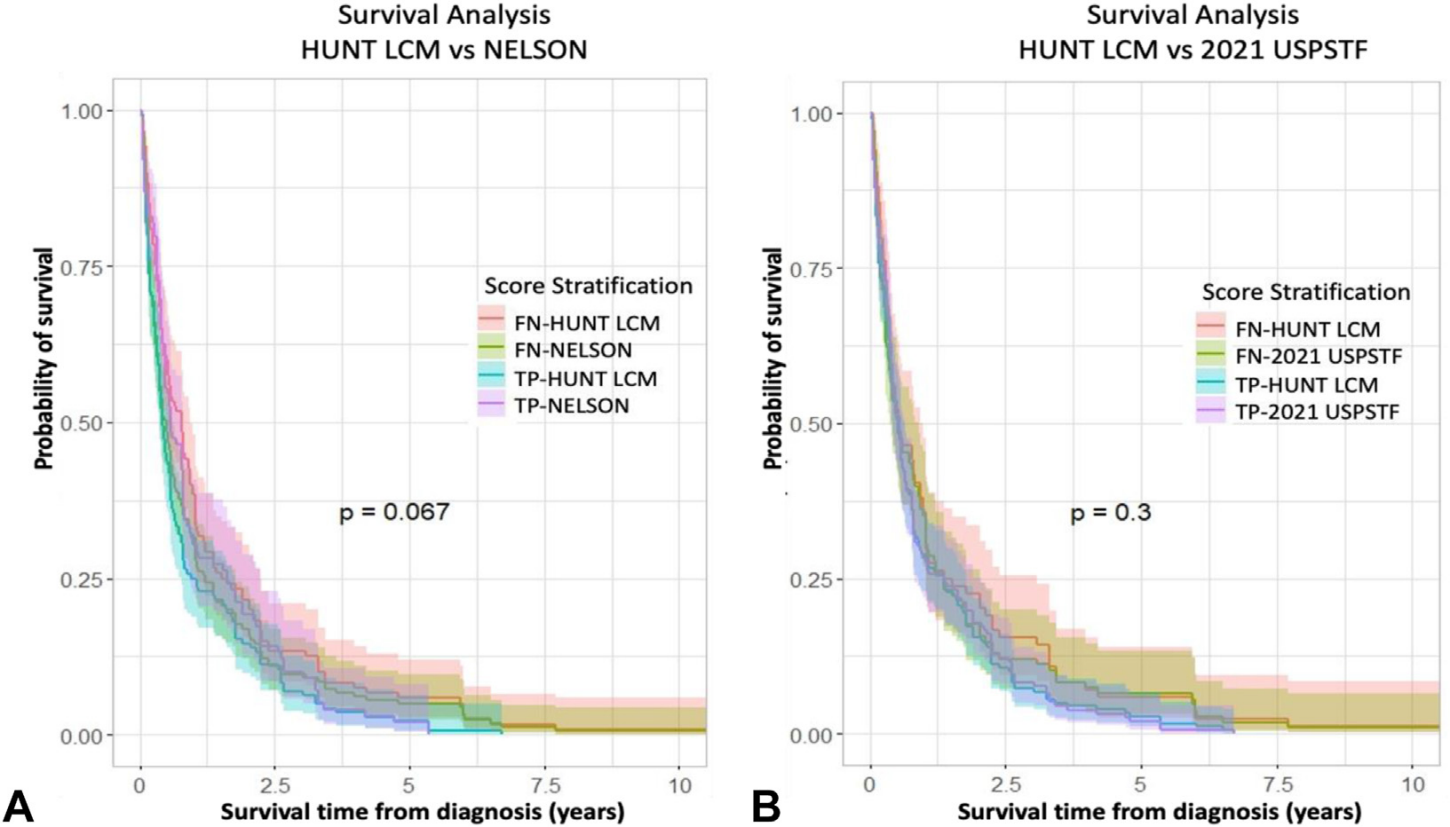
Overall survival from the time of diagnosis to death. Insignificant differences in survival among the individuals in CONOR selected by the HUNT LCM that developed lung cancer in 6 years compared with the TP patients within 6 years predicted by the NELSON and 2021 USPSTF criteria. Kaplan-Meier curves: (*A*) HUNT LCM versus NELSON criteria. (*B*) HUNT LCM vs 2021 USPSTF criteria. NELSON criteria include the following: (1) age between 50 to 74 years old; (2) greater than15 cigarettes per day for more than 25 years or greater than 10 cigarettes per day for more than 30 years; and quit smoking less than or equal to 10 years. The 2021 USPSTF criteria include the following: (1) age between 50 to 80 years old; (2) at least 20 pack-years; and (3) currently smoking or quit smoking less than 15 years. HUNT LCM, HUNT Lung Cancer Model; CONOR, Cohort of Norway; USPSTF United States Preventive Services Task Force; FN, false-negative; FP, false-positive; TN, true negative; TP, true positive.

When compared with the NELSON and 2021 USPSTF criteria, there were no significant differences found in median survival from diagnosis of patients that developed LC within 6 years predicted by the HUNT LCM (10.9 versus 13.1 mo, *p* = 0.067; and 12.0 versus 12.2 mo, *p* = 0.3, respectively) (Fig. 3).

### Predictive Performance of the HUNT LCM of the Top 16th Percentile Risk Scores Versus NELSON and 2021 USPSTF Criteria

The top 16th percentile risk threshold for LC risk computed on the HUNT2 cohort was 0.98% risk of LC diagnosis in 6 years. When this threshold was applied to stratify high and low risk, the HUNT LCM identified 6264 (14.0%) ever-smokers as high-risk for LC (Supplementary Table 9). The age distribution in this subcohort ranged from 40 to older than 90 years and 95% of the selected patients were between 50 to 80 years (Supplementary Table 10). The model exhibited increased predictive performance by identifying 157 LCs in 6 years corresponding to a sensitivity of 70.7%, increasing LC detection rate with 89.2% and 18.9% compared with the NELSON and 2021 USPSTF criteria, respectively (Supplementary Table 11). The sensitivity of the HUNT LCM increased significantly (*p* < 0.001), but with the cost of a lower, but still relatively high, specificity (86.3%), and the PPV of the HUNT LCM remained similar to the two criteria (Supplementary Table 11).

Among the 157 true-positive patients predicted by the HUNT LCM, 74 and 120 of these were cases predicted by the NELSON and 2021 USPSTF criteria, respectively, as well (Supplementary Fig. 2). Nine and 12 LC cases predicted by NELSON and 2021 USPSTF criteria, respectively, were not predicted by the HUNT LCM when the top 16th percentile was applied as the risk threshold (Supplementary Fig. 2).

## Discussion

In previous work, the HUNT LCM was found to be more sensitive and effective compared with the NLST criteria in LC risk stratification.^11^ In the present study, we further compared the HUNT LCM lung cancer predictive performance with the NELSON and 2021 USPSTF criteria. The results are significantly in favor of HUNT LCM, both in LC risk assessment and efficiency in detecting a single LC case.

**Figure 4:**
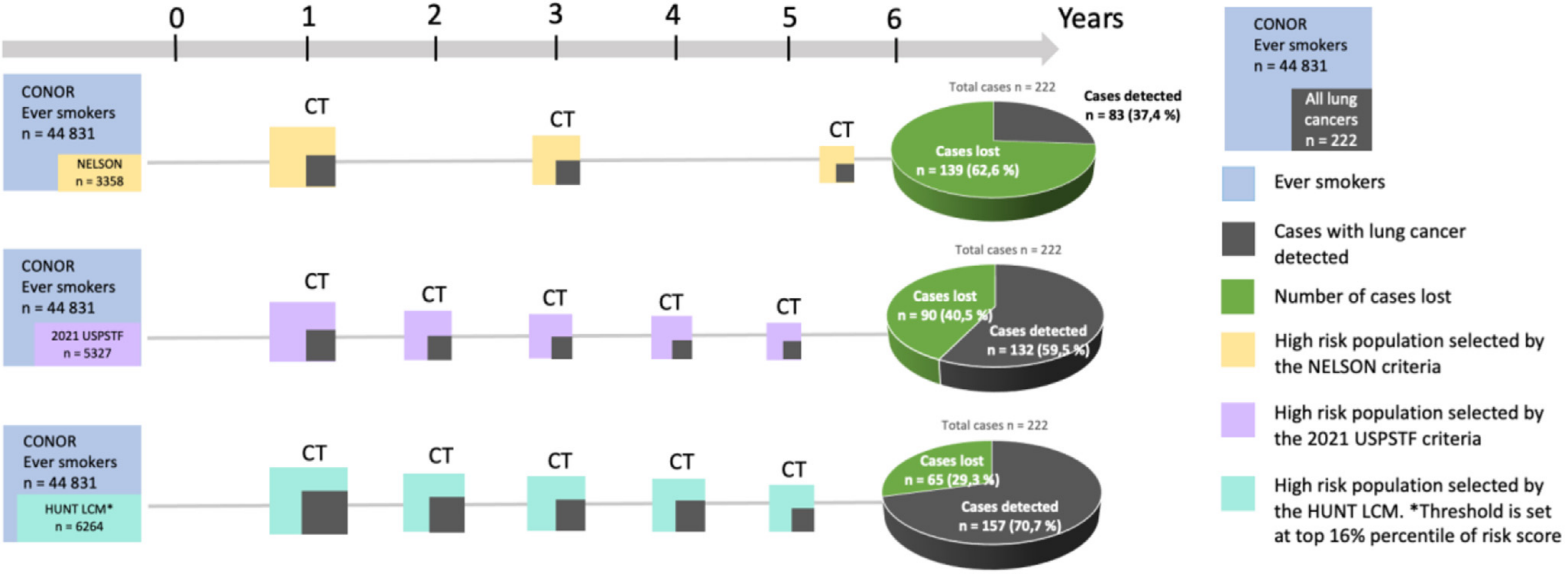
Improving lung cancer prediction of high-risk individuals before CT screening. Cartoon comparing the HUNT LCM against the NELSON and 2021 USPSTF criteria in the CONOR data set of 44831 ever-smokers, in which 222 individuals developed lung cancer within 6 years. The dark gray boxes depict diagnosed lung cancers within the 6 years. The CT boxes on the timeline depict hypothetical screenings. NELSON criteria include the following: (1) age between 50 to 74 years old; (2) greater than15 cigarettes per day for more than 25 years or greater than 10 cigarettes per day for more than 30 years; and quit smoking less than or equal to 10 years. The 2021 USPSTF criteria include the following: (1) age between 50 to 80 years old; (2) at least 20 pack-years; and (3) currently smoking or quit smoking less than 15 years. The HUNT LCM top 16th risk score in the HUNT2 represents the absolute cutoff 0.985% risk in 6 years. CT, computer tomography; HUNT LCM, HUNT Lung Cancer Model. CONOR, Cohort of Norway; NELSON, Nederlands-Leuvens Longkanker Screenings Onderzoek; USPSTF, United States Preventive Services Task Force.

The main reason to search for risk prediction models is to reliably predict those at true high risk. Previous studies from the United States and Norway reported that the NLST criteria excluded three-quarters of all ever-smokers developing LC.^5^^,^^11^ Our study of ever-smokers in this large Norwegian population study found that the NELSON and 2021 USPSTF would exclude 62.6% and 40.5% of future LC cases, respectively, reaffirming the improvement potential of the current selection criteria. The HUNT LCM outperformed the 2021 USPSTF and NELSON criteria regarding sensitivity, PPV, negative predictive value, and NNS. For a fair comparison, when the same number of individuals were selected as the NELSON and 2021 USPSTF criteria sets, the HUNT LCM identified 41.0% and 12.1% additional cases in the CONOR ever-smokers, respectively (Table 2 and Supplementary Table 2). When the top 16th percentile is applied as the cutoff for risk stratification^11^^,^^15^ the HUNT LCM identified 89.2% and 18.9% more cases in CONOR within 6 years compared with the NELSON and 2021 USPSTF criteria, respectively (Fig. 4). Furthermore, the model’s effectiveness in selecting high-risk individuals for LC screening was significantly better compared with the categorical approaches measured by the number of screenings required per cancer detected (NNS) (Fig. 2).

The present results are in line with our previous work and others,^11^, ^12^, ^13^^,^^16^^,^^17^ supporting a risk-based approach over a categorical approach in LC screening. Our results are similar to the recently published interim results of the International Lung Cancer Screening Trial, which made similar comparisons and found superior performance of the risk prediction model PLCO_m2012_ in LC prediction within 6 years, but that comparison was against the 2013 USPSTF criteria.^13^ When the upper age limits of the NELSON and 2021 USPSTF criteria are employed as cutoffs (<75 and <81 y of age, respectively), the HUNT LCM still performs better than both the criteria (Supplementary Tables 3 and 5). A similar significant superior performance of the HUNT LCM was seen when restricting the age as the 2021 USPSTF criteria at 50 to 80 years of age, increasing sensitivity from 65.7 to 73.6% (Supplementary Tables 7 and 8). Using age limits to the risk model can be problematic as there are high-risk individuals both those below the age of 50 and above 80 years. According to our results, few of the individuals that were included below 50 years of age and developed LC within 6 years were predicted by the HUNT LCM. On the upper limit, very few of those included above the age of 80 developed LC within 6 years (Supplementary Table 10). Thus, one can argue that if one should implement an age criterion with the HUNT LCM, a cutoff of 50 to 80 years of age in line with the 2021 USPSTF criteria could be used, retaining a high overall performance. Regarding the prognosis of those selected by the clinical criteria versus the HUNT LCM, there is no difference in median survival from diagnosis to death between patients with LC detected in 6 years, thus one cannot argue that the model selects people with higher mortality after LC diagnosis (Fig. 3). Despite growing evidence that risk prediction models perform better in LC risk stratification, there is still a reluctance to adopt risk models for patient selection for CT screening.^7^ Whereas some countries have adopted risk prediction models in their screening programs, there is still discussion in several European countries. One argument against risk models is that they have better predictive performance because of the fact that the individuals selected predominantly are of older age leading potentially to fewer life years gained.^7^^,^^18^^,^^19^ This argument implies difficult ethical considerations, because an 80-year-old person will suffer as much as a younger person if the cancer metastasizes, and the need for expensive treatments and hospital stays will not be different. His or her suffering can be avoided by early detection and surgery or curative stereotactic radiation treatment. If an elderly person is at high risk and fit enough for surgery or radiotherapy, is it ethical to exclude this person from screening because of age?

One of the important advantages of using a risk model over the categorical approaches such as the NELSON, NLST, and USPSTF criteria is that the threshold for high or low risk is adjustable and can be set to reflect the screening capacity of each country and health system. On the basis of the recommendations from Royston et al.^11^^,^^15^ we applied the top 16th percentile of the discovery cohort, equal to 0.98% risk in 6 years, in the Norwegian cohort (6264 patients, predicting 26% more than the 2021 USPSTF criteria). Here, we found that a threshold including slightly more individuals significantly increased sensitivity in an efficient way, and detected more cases in the younger age group 40 to 60 years (Supplementary Table 10).

The study has several strengths worth noting. First, the CONOR is a national database including populations from various parts of Norway, both rural and urban, and therefore, not confined to certain social classes.^14^ Second, the sample size of CONOR, the long follow-up time, and the prospectively collected clinical data increase the statistical robustness of the findings. Third, other models such as the PLCO _m2012_ use additional culture-specific or diagnosis-based factors that may be susceptible to bias (education, ethnicity, history of chronic obstructive pulmonary disease, or family history of LC).^12^ In contrast, the HUNT LCM uses easily retrievable variables independent of these factors. Among these variables, we recognize that the two variables “symptoms of daily cough in periods of the year” and “hours of indoor smoke” are often unavailable in databases from other countries, making it difficult to compare with our model. However, this is only an issue in research settings in which databases are used to test the different risk prediction models. In clinical settings, this is less of an issue, because one needs to retrieve all the variables directly from each participant and not from such databases, and the variables in the HUNT LCM can readily be answered. However, although the two variables “symptoms of daily cough in periods of the year” and “hours of indoor smoke” are easily answered by individuals, we acknowledge that they are not as easy to answer precisely as the other variables in the model, and these two are often unavailable in databases from other countries. In case one does not have access to these two variables, one may use the HUNT LCM omitting these variables, or use our “reduced” HUNT model that was published previously.^12^ Fourth, in screening detected cancers, the proportion of indolent LCs was found to be greater than 18%.^20^^,^^21^ Clinically detected LCs are rarely indolent. As there is no screening program in Norway, all LC in the CONOR were clinically detected. The survival analysis revealed a median survival of 10 to 12 months from diagnosis (Fig. 3) indicating that the cases detected by the HUNT LCM are mostly nonindolent. Therefore, the high predictive performance of the HUNT LCM and the relatively short overall survival of those diagnosed indicate that a screening program using the HUNT LCM will translate into lower mortality compared with the NELSON and 2021 USPSTF criteria.

Several limitations of the HUNT LCM have already been discussed in our previous publication of the model,^11^ including the lack of information on some known LC risk predictors (history of chronic obstructive pulmonary disease, occupational exposure to asbestos or radon, and heredity) in the HUNT2 data set, which the HUNT LCM was developed from and trained on. Furthermore, we note that the model has not been validated in external cohorts in populations outside Scandinavia. We also recognize a potential bias issue in the validation cohort because of filtering out incomplete data.

In conclusion, the validated HUNT LCM had superior performance compared with the NELSON and 2021 USPSTF criteria. The present study suggests that the HUNT LCM improves the prediction of LC diagnosis and is more efficient, with significantly fewer individuals selected to predict one LC. This is important for policymakers to plan high-value (sustainable) health care programs. Furthermore, the simplicity and efficacy of HUNT LCM as exhibited by the published online calculator^22^ makes it easy to apply in clinical practice. The HUNT LCM may be used as a clinical tool for LC screening selection. Further validation in other populations is ongoing. Though evidence supports risk-based screening over categorical screening in LC, it still remains unclear which risk prediction model should be used. Therefore, it is important to address this question in future studies. This will also help policymakers in their work of implementing risk prediction models in LC screening programs. A prospective study comparing the HUNT LCM and one of the other risk prediction models is ongoing in Norway.

## CRediT Authorship Contribution Statement

**Olav Toai Duc Nguyen:** Conceptualization, Investigation, Writing – original draft, Writing – review & editing, Visualization.

**Ioannis Fotopoulos:** Formal analysis, Validation, Data Curation, Writing – review & editing, Visualization

**Maria Markaki:** Formal analysis, Validation, Writing – review & editing

**Ioannis Tsamardinos:** Methodology, Writing – review & editing

**Vincenzo Lagani:** Methodology, Writing – review & editing.

**Oluf Dimitri Røe:** Conceptualization, Methodology, Investigation, Writing – Original Draft, Writing – Review & Editing, Visualization, Supervision, Project administration.

## Disclosure

The authors declare no conflict of interest.
