# Supplementary material for: Improving Lung Cancer Screening Selection: The HUNT Lung Cancer Risk Model for Ever-Smokers Versus the NELSON and 2021 United States Preventive Services Task Force Criteria in the Cohort of Norway: A Population-Based Prospective Study
Source: JTO Clin Res Rep. 2024 Mar 5;5(4):100660. doi: 10.1016/j.jtocrr.2024.100660 (PMC10998221; doi:10.1016/j.jtocrr.2024.100660)
Supplement: Supplementary Material [file mmc1.docx]

**Improving lung cancer screening selection: the HUNT Lung Cancer Risk Model for ever-smokers versus the NELSON and 2021 USPSTF criteria in the Cohort of Norway (CONOR), a Population-based Prospective Study**

**Olav Toai Duc Nguyen, Ioannis Fotopoulos, Maria Markaki, Ioannis Tsamardinos, Vincenzo Lagani, Oluf Dimitri Røe**

**Supplementary Material**

**Supplementary Methods**

**Model Comparison**

The predictive performance of the HUNT LCM was also tested using the top 16th percentile computed on the ever-smokers in the HUNT2 cohort (risk scores >0.98% risk in six years). The top 16th percentile risk was used to equal the risk threshold employed in our original paper.^1^ This threshold is not optimizing any trade-off in our data according to some selective results, but it was used based on the work by Royston et al.^2^ In short Royston states: “For a given number of groups, the method is designed to minimise the loss of information that occurs with grouping. The required cut-points are the 16th, 50th and 84th centiles of the continuous variable, here the prognostic index in the derivation dataset. On a standard Normal scale, these correspond to 0 and approximately ±1, i.e. mean ± 1SD.”^2^ This threshold was applied in CONOR to stratify subjects in high- and low-risk categories according to the HUNT LCM.

**Supplementary Results**

**Supplementary Table 1.** Age distribution at inclusion in the CONOR study of those that developed lung cancer within six years.

| **CONOR population** | |
| --- | --- |
| **Age intervals** | **Lung cancer cases within six years**  **N** |
| **<40** | 1 |
| **40-49** | 19 |
| **50-59** | 38 |
| **60-69** | 82 |
| **70-79** | 76 |
| **80-90** | 6 |
| **>90** | 0 |
| **Total** | 222 |

**Supplementary Table 2**. Comparison of the HUNT Lung Cancer Model (HUNT LCM) against the 2021 USPSTF criteria. The comparison is performed by considering the number of individuals selected by the 2021 USPSTF criteria, i.e., n=5327 on the CONOR cohort (n=44831).

2021 USPSTF criteria: age between 50 to 80 years old, at least 20 pack-years, and currently smoking or quit smoking <15 years.

FN, false negative; FP, false positive; NPV, negative predictive value; PPV, positive predictive value; TN, true negative; TP, true positive lung cancer cases.

^a^P-value >.05, versus 2021 USPSTF

^b^P-value <.05, versus 2021 USPSTF

^c^P-value <.01, versus 2021 USPSTF

|  | **CONOR ever-smokers** | | | |
| --- | --- | --- | --- | --- |
|  | **Lung cancers (N=222)** | **No lung cancer (N=44609)** | **Total (N=44831)** | **Predictive value** |
| **2021 USPSTF** |  |  |  |  |
| Criteria positive | 132 TP (2.5%) | 5195 FP (97.5%) | 5327 | PPV 2.5% |
| Criteria negative | 90 FN (0.2%) | 39414 TN (99.8%) | 39504 | NPV 99.8% |
| Sensitivity | 59.5% |  |  |  |
| Specificity |  | 88.4% |  |  |
| **HUNT LCM** |  |  |  |  |
| Criteria positive | 148 TP (2.8%) | 5179 FP (97.2%) | 5327 | PPV 2.8%^b^ |
| Criteria negative | 74 FN (0.2%) | 39430 TN (99.8%) | 39504 | NPV 99.8%^c^ |
| Sensitivity | 66.7%^b^ |  |  |  |
| Specificity |  | 88.4%^a^ |  |  |

**Supplementary Table 3.** Lung cancer predictive performance of the HUNT Lung Cancer Model (HUNT LCM) versus using the upper age criterion (<75 years of age) of the NELSON criteria as a cutoff. Individuals ≥75 years of age are excluded from the analysis.
FN, false negative; FP, false positive; NPV, negative predictive value; PPV, positive predictive value; TN, true negative; TP, true positive lung cancer cases.
NELSON criteria: age between 50 to 74 years old, >15 cigarettes per day for >25 years or >10 cigarettes per day for >30 years, quit smoking ≤10 years.
^a^P-value >.05, versus NELSON
^b^P-value <.05, versus NELSON
^c^P-value <.01, versus NELSON

|  | **CONOR ever-smokers** | | | |
| --- | --- | --- | --- | --- |
|  | **Lung cancers (N=172)** | **No lung cancer (N=41864)** | **Total (N=42036)** | **Predictive value** |
| **NELSON** |  |  |  |  |
| Criteria positive | 83 TP (2.5%) | 3275 FP (97.5%) | 3358 | PPV 2.5% |
| Criteria negative | 89 FN (0.2%) | 38589 TN (99.8%) | 38678 | NPV 99.8% |
| Sensitivity | 48.3% |  |  |  |
| Specificity |  | 92.2% |  |  |
| **HUNT LCM^NELSON <75^** |  |  |  |  |
| Criteria positive | 98 TP (2.9%) | 3260 FP (97.1%) | 3358 | PPV 2.9%^b^ |
| Criteria negative | 74 FN (0.2%) | 38604 TN (99.8%) | 38678 | NPV 99.8%^c^ |
| Sensitivity | 57.0%^b^ |  |  |  |
| Specificity |  | 92.2%^a^ |  |  |

**Supplementary Table 4.** Lung cancer predictive performance of the HUNT Lung Cancer Model (HUNT LCM) versus using the upper age criterion (<81 years of age) of the 2021 USPSTF criteria as a cutoff. Individuals ≥81 years of age are excluded from the analysis.
FN, false negative; FP, false positive; NPV, negative predictive value; PPV, positive predictive value; TN, true negative; TP, true positive lung cancer cases.
2021 USPSTF criteria: age between 50 to 80 years old, at least 20 pack-years, and currently smoking or quit smoking <15 years.
^a^P-value >.05, versus 2021 USPSTF
^b^P-value <.05, versus 2021 USPSTF
^c^P-value <.01, versus 2021 USPSTF

|  | **CONOR ever-smokers** | | | |
| --- | --- | --- | --- | --- |
|  | **Lung cancers (N=221)** | **No lung cancer (N=44466)** | **Total (N=44687)** | **Predictive value** |
| **2021 USPSTF** |  |  |  |  |
| Criteria positive | 132 TP (2.5%) | 5195 FP (97.5%) | 5327 | PPV 2.5% |
| Criteria negative | 89 FN (0.2%) | 39271 TN (99.8%) | 39360 | NPV 99.8% |
| Sensitivity | 59.7% |  |  |  |
| Specificity |  | 88.3% |  |  |
| **HUNT LCM^2021USPSTF <81^** |  |  |  |  |
| Criteria positive | 148 TP (2.8%) | 5179 FP (97.2%) | 5327 | PPV 2.8%^b^ |
| Criteria negative | 73 FN (0.2%) | 39287 TN (99.8%) | 39360 | NPV 99.8%^c^ |
| Sensitivity | 67.0%^c^ |  |  |  |
| Specificity |  | 88.4%^a^ |  |  |

**Supplementary Table 5.** Age distribution at inclusion in CONOR of eligible population and lung cancer cases (true positives) within six years predicted by the NELSON criteria and the HUNT LCM when equal numbers of individuals are considered.
NNS, number needed to screen to identify one case of lung cancer.

| **CONOR population** | **NELSON** | | | **HUNT LCM** | | |
| --- | --- | --- | --- | --- | --- | --- |
| **Age intervals** | **High risk**  **N** | **True positive**  **N** | **NNS** | **High risk**  **N** | **True positive**  **N** | **NNS** |
| **<40** | 0 | 0 | - | 0 | 0 | - |
| **40-49** | 0 | 0 | - | 9 | 0 | - |
| **50-59** | 1566 | 25 | 62 | 493 | 12 | 41 |
| **60-69** | 1535 | 46 | 33 | 1374 | 47 | 29 |
| **70-79** | 257 | 12 | 21 | 1389 | 54 | 25 |
| **80-90** | 0 | 0 | - | 90 | 4 | 22 |
| **>90** | 0 | 0 | - | 3 | 0 | - |
| **Total** | 3358 | 83 | 40 | 3358 | 117 | 28 |

**Supplementary Table 6.** Age distribution at inclusion in CONOR eligible population and lung cancer cases (true positives) within six years predicted by the 2021 USPSTF criteria and the HUNT LCM when equal numbers of individuals are considered.

NNS, number needed to screen to identify one case of lung cancer.

| **CONOR population** | **2021 USPSTF** | | | **HUNT LCM** | | |
| --- | --- | --- | --- | --- | --- | --- |
| **Age intervals** | **High risk**  **N** | **True positive**  **N** | **NNS** | **High risk** | **True positive** | **NNS** |
| **<40** | 0 | 0 | - | 0 | 0 | - |
| **40-49** | 0 | 0 | - | 81 | 0 | - |
| **50-59** | 1932 | 25 | 77 | 1116 | 19 | 58 |
| **60-69** | 2223 | 65 | 34 | 2149 | 62 | 34 |
| **70-79** | 1154 | 40 | 28 | 1858 | 61 | 30 |
| **80-90** | 18 | 2 | 9 | 120 | 6 | 20 |
| **>90** | 0 | 0 |  | 3 | 0 |  |
| **Total** | 5327 | 132 | 40 | 5327 | 148 | 36 |

**Supplementary Table 7.** Lung cancer predictive performance of the HUNT Lung Cancer Model (HUNT LCM) versus the NELSON criteria using the age cutoff of 50-80. Individuals <50 years and ≥81 years of age are excluded from the analysis.
FN, false negative; FP, false positive; NPV, negative predictive value; PPV, positive predictive value; TN, true negative; TP, true positive lung cancer cases.
NELSON criteria: age between 50 to 74 years old, >15 cigarettes per day for >25 years or >10 cigarettes per day for >30 years, quit smoking ≤10 years.
^a^P-value >.05, versus NELSON
^b^P-value <.01, versus NELSON

|  | **CONOR ever-smokers** | | | |
| --- | --- | --- | --- | --- |
|  | **Lung cancers (N=201)** | **No lung cancer (N=15654)** | **Total (N=15855)** | **Predictive value** |
| **NELSON** |  |  |  |  |
| Criteria positive | 83 TP (2.5%) | 3275 FP (97.5%) | 3358 | PPV 2.47% |
| Criteria negative | 118 FN (0.9%) | 12379 TN (99.1%) | 12430 | NPV 99.1% |
| Sensitivity | 41.3% |  |  |  |
| Specificity |  | 79.0% |  |  |
| **HUNT LCM^50-80^** |  |  |  |  |
| Criteria positive | 121 TP (3.6%) | 3237 FP (96.4%) | 3358 | PPV 3.60%^b^ |
| Criteria negative | 80 FN (0.6%) | 12417 TN (99.4%) | 12430 | NPV 99.4%^b^ |
| Sensitivity | 60.2%^b^ |  |  |  |
| Specificity |  | 79.3%^a^ |  |  |

**Supplementary Table 8.** Lung cancer predictive performance of the HUNT Lung Cancer Model (HUNT LCM) versus using the same age cutoff as the 2021 USPSTF criteria (50-80 years of age). Individuals <50 years and ≥81 years of age are excluded from the analysis.
FN, false negative; FP, false positive; NPV, negative predictive value; PPV, positive predictive value; TN, true negative; TP, true positive lung cancer cases.
2021 USPSTF criteria: age between 50 to 80 years old, at least 20 pack-years, and currently smoking or quit smoking <15 years.
^a^P-value >.05, versus 2021 USPSTF
^b^P-value <.05, versus 2021 USPSTF

|  | **CONOR ever-smokers** | | | |
| --- | --- | --- | --- | --- |
|  | **Lung cancers (N=201)** | **No lung cancer (N=15654)** | **Total (N=15855)** | **Predictive value** |
| **2021 USPSTF** |  |  |  |  |
| Criteria positive | 132 TP (2.5%) | 5195 FP (97.5%) | 5313 | PPV 2.5% |
| Criteria negative | 69 FN (0.7%) | 10459 TN (99.3%) | 10475 | NPV 99.3% |
| Sensitivity | 65.7% |  |  |  |
| Specificity |  | 66.8% |  |  |
| **HUNT LCM^50-80^** |  |  |  |  |
| Criteria positive | 148 TP (2.7%) | 5179 FP (97.3%) | 5313 | PPV 2.8%^b^ |
| Criteria negative | 53 FN (0.5%) | 10475 TN (99.5%) | 10475 | NPV 99.5%^b^ |
| Sensitivity | 73.6%^b^ |  |  |  |
| Specificity |  | 66.9%^a^ |  |  |

**Supplementary Table 9.** Performance of the HUNT Lung Cancer Model (HUNT LCM) in predicting lung cancer diagnosis within six years on the CONOR ever-smokers (n=44831). The top 16th percentile risk threshold for lung cancer risk computed on the HUNT2 cohort was 0.98% in six years. This threshold was used as a cutoff to separate high and low risk subjects in CONOR.
FN, false negative; FP, false positive; NPV, negative predictive value; PPV, positive predictive value; TN, true negative; TP, true positive lung cancer cases.

|  | **CONOR ever-smokers** | | | |
| --- | --- | --- | --- | --- |
|  | **Lung cancers (N=222)** | **No lung cancer (N=44609)** | **Total (N=44831** | **Predictive value** |
| **HUNT LCM** |  |  |  |  |
| Criteria positive | 157 TP (2.5%) | 6107 FP (97.5%) | 6264 | PPV 2.5% |
| Criteria negative | 65 FN (0.2%) | 38502 TN (99.8%) | 38567 | NPV 99.8% |
| Sensitivity | 70.7% |  |  |  |
| Specificity |  | 86.3% |  |  |

**Supplementary Table 10.** Age distribution at inclusion in CONOR of eligible population and lung cancer cases (true positive) within six years predicted by the HUNT Lung Cancer Model (HUNT LCM), risk score of >0.98% (corresponding to top 16th percentile risk threshold) in six years.
NNS, number needed to screen to identify one case of lung cancer.

| **CONOR population** | **HUNT LCM >0.98% risk score** | | |
| --- | --- | --- | --- |
| **Age intervals** | **High risk**  **N** | **True positive**  **N** | **NNS** |
| **<40** | 0 | 0 | - |
| **40-49** | 171 | 2 | 85 |
| **50-59** | 1402 | 22 | 63 |
| **60-69** | 2475 | 63 | 39 |
| **70-79** | 2080 | 64 | 32 |
| **80-90** | 133 | 6 | 22 |
| **>90** | 3 | 0 | - |
| **Total** | 6264 | 157 | 40 |

**Supplementary Table 11**. Comparison of the HUNT Lung Cancer Model (HUNT LCM) performance when the top 16th percentile risk threshold is applied against the NELSON and 2021 USPSTF criteria on the CONOR ever-smokers. The top 16th percentile risk threshold for lung cancer risk computed on the HUNT2 cohort was 0.98% in six years. This threshold was used as a cutoff to separate high and low risk subjects in CONOR.
The comparison is performed by considering different number of individuals selected by the NELSON (n=3358), 2021 USPSTF criteria (n=5327) and HUNT LCM (n=6264) on the CONOR ever-smokers cohort (n=44831).
NELSON criteria: age between 50 to 74 years old, >15 cigarettes per day for >25 years or >10 cigarettes per day for >30 years, quit smoking ≤10 years.
2021 USPSTF criteria: age between 50 to 80 years old, at least 20 pack-years, and currently smoking or quit smoking <15 years.
FN, false negative; FP, false positive; NPV, negative predictive value; PPV, positive predictive value; TN, true negative; TP, true positive.
^a^P-value >.05, versus NELSON and 2021 USPSTF
^b^P-value <.01, versus NELSON and 2021 USPSTF

|  | **CONOR ever-smokers** | | | |
| --- | --- | --- | --- | --- |
|  | **Lung cancers (N=222)** | **No lung cancer (N=44609)** | **Total (N=44831)** | **Predictive value** |
| **NELSON** |  |  |  |  |
| Criteria positive | 83 TP (2.5%) | 3275 FP (97.5%) | 3358 | PPV 2.5% |
| Criteria negative | 139 FN (0.3%) | 41334 TN (99.7%) | 41473 | NPV 99.7% |
| Sensitivity | 37.4% |  |  |  |
| Specificity |  | 92.7% |  |  |
| **2021 USPSTF** |  |  |  |  |
| Criteria positive | 132 TP (2.5%) | 5195 FP (97.5%) | 5327 | PPV 2.5% |
| Criteria negative | 90 FN (0.2%) | 39414 TN (99.8%) | 39504 | NPV 99.8% |
| Sensitivity | 59.5% |  |  |  |
| Specificity |  | 88.4% |  |  |
| **HUNT LCM^top 16th percentile^** |  | | | |
| Criteria positive | 157 TP (2.5%) | 6107 FP (97.5%) | 6264 | PPV 2.5%^a^ |
| Criteria negative | 65 FN (0.2%) | 38502 TN (99.8%) | 38567 | NPV 99.8%^b^ |
| Sensitivity | 70.7%^b^ |  |  |  |
| Specificity |  | 86.3%^b^ |  |  |

**
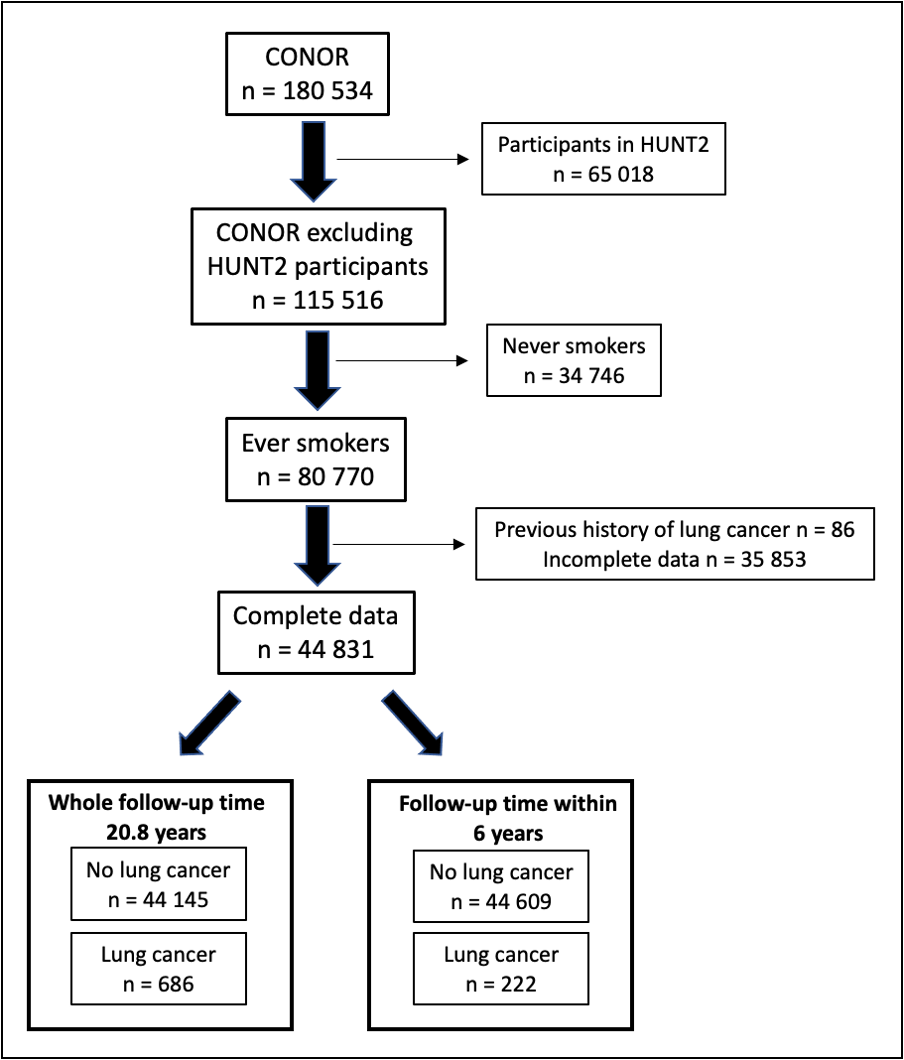
**

**Supplementary Figure 1.** Consort flow diagram for inclusion.


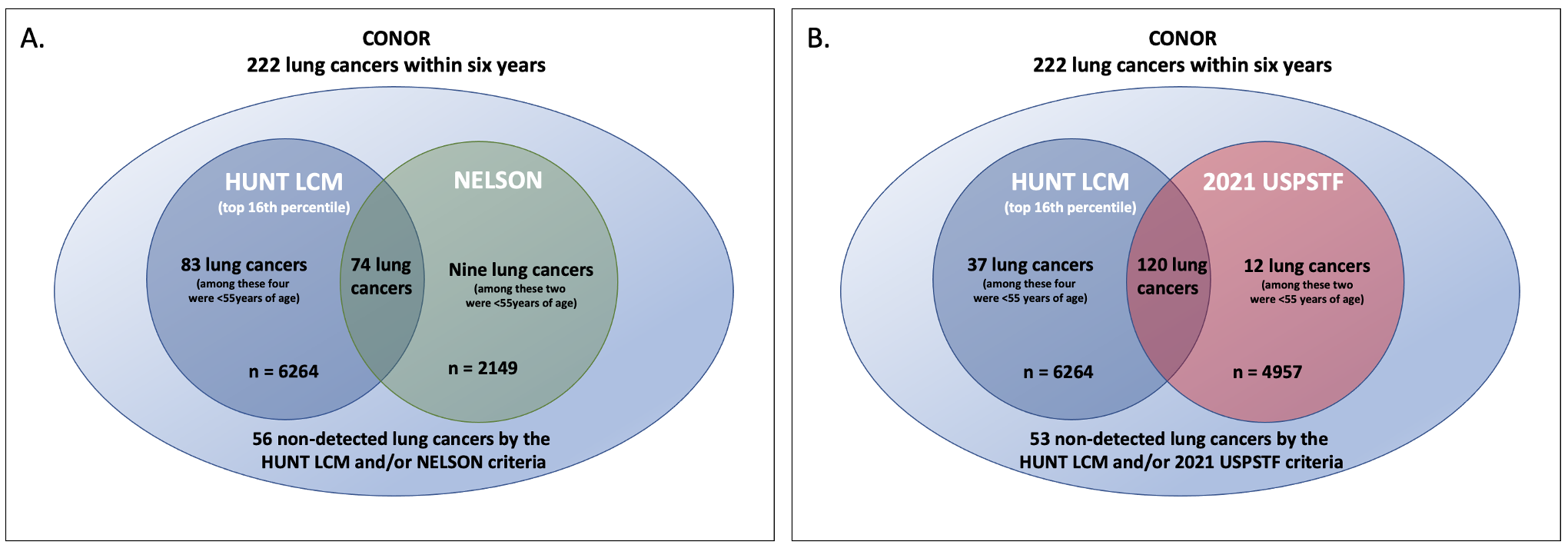


**Supplementary Figure 2.** Venn diagram describing the distribution of lung cancer cases within six years by the HUNT Lung Cancer Model (HUNT LCM) (top 16th percentile) and the criteria A. NELSON and B. 2021 USPSTF. Of the lung cancer cases in CONOR that were predicted by the HUNT LCM and not by the NELSON and 2021 USPSTF criteria, four of these were below the age of 55, while the two criteria predicted both two lung cancer cases below 55 years of age that were not predicted by the HUNT LCM (P=0.16).

**References**

1. Markaki M, Tsamardinos I, Langhammer A, Lagani V, Hveem K, Roe OD. A Validated Clinical Risk Prediction Model for Lung Cancer in Smokers of All Ages and Exposure Types: A HUNT Study. EbioMedicine. 2018;31:36-46.

2. Royston P, Altman DG. External validation of a Cox prognostic model: principles and methods. BMC Med Res Methodol. 2013;13:33.
